# The structure of *Haemophilus influenzae* prephenate dehydrogenase suggests unique features of bifunctional TyrA enzymes

**DOI:** 10.1107/S1744309110021688

**Published:** 2010-07-31

**Authors:** Hsiu-Ju Chiu, Polat Abdubek, Tamara Astakhova, Herbert L. Axelrod, Dennis Carlton, Thomas Clayton, Debanu Das, Marc C. Deller, Lian Duan, Julie Feuerhelm, Joanna C. Grant, Anna Grzechnik, Gye Won Han, Lukasz Jaroszewski, Kevin K. Jin, Heath E. Klock, Mark W. Knuth, Piotr Kozbial, S. Sri Krishna, Abhinav Kumar, David Marciano, Daniel McMullan, Mitchell D. Miller, Andrew T. Morse, Edward Nigoghossian, Linda Okach, Ron Reyes, Henry J. Tien, Christine B. Trame, Henry van den Bedem, Dana Weekes, Qingping Xu, Keith O. Hodgson, John Wooley, Marc-André Elsliger, Ashley M. Deacon, Adam Godzik, Scott A. Lesley, Ian A. Wilson

**Affiliations:** a Stanford Synchrotron Radiation Lightsource, SLAC National Accelerator Laboratory, Menlo Park, CA, USA; b Joint Center for Structural Genomics, http://www.jcsg.org, USA; c Protein Sciences Department, Genomics Institute of the Novartis Research Foundation, San Diego, CA, USA; d Center for Research in Biological Systems, University of California, San Diego, La Jolla, CA, USA; e Department of Molecular Biology, The Scripps Research Institute, La Jolla, CA, USA; f Program on Bioinformatics and Systems Biology, Sanford–Burnham Medical Research Institute, La Jolla, CA, USA; g Photon Science, SLAC National Accelerator Laboratory, Menlo Park, CA, USA

**Keywords:** tyrosine biosynthesis, prephenate, chorismate, *Haemophilus influenzae*, structural genomics

## Abstract

Chorismate mutase/prephenate dehydrogenase from *Haemophilus influenzae* Rd KW20 is a bifunctional enzyme that catalyzes the rearrangement of chorismate to prephenate and the NAD(P)^+^-dependent oxidative decarboxyl­ation of prephenate to 4-hydroxyphenylpyruvate in tyrosine biosynthesis. The crystal structure of the prephenate dehydrogenase component (*Hinf*PDH) of the TyrA protein from *H. influenzae* Rd KW20 in complex with the inhibitor tyrosine and cofactor NAD^+^ has been determined to 2.0 Å resolution. *Hinf*PDH is a dimeric enzyme, with each monomer consisting of an N-terminal α/β dinucleotide-binding domain and a C-terminal α-helical dimerization domain. The structure reveals key active-site residues at the domain interface, including His200, Arg297 and Ser179 that are involved in catalysis and/or ligand binding and are highly conserved in TyrA proteins from all three kingdoms of life. Tyrosine is bound directly at the catalytic site, suggesting that it is a competitive inhibitor of *Hinf*PDH. Comparisons with its structural homologues reveal important differences around the active site, including the absence of an α–β motif in *Hinf*PDH that is present in other TyrA proteins, such as *Synechocystis* sp. arogenate dehydrogenase. Residues from this motif are involved in discrimination between NADP^+^ and NAD^+^. The loop between β5 and β6 in the N-terminal domain is much shorter in *Hinf*PDH and an extra helix is present at the C-terminus. Furthermore, *Hinf*PDH adopts a more closed conformation compared with TyrA proteins that do not have tyrosine bound. This conformational change brings the substrate, cofactor and active-site residues into close proximity for catalysis. An ionic network consisting of Arg297 (a key residue for tyrosine binding), a water molecule, Asp206 (from the loop between β5 and β6) and Arg365′ (from the additional C-terminal helix of the adjacent monomer) is observed that might be involved in gating the active site.

## Abstract 2

The crystal structure of the prephenate dehydrogenase component of the bifunctional *H. influenzae* TyrA reveals unique structural differences between bifunctional and monofunctional TyrA enzymes.

## 1. Introduction

**Figure 1:**
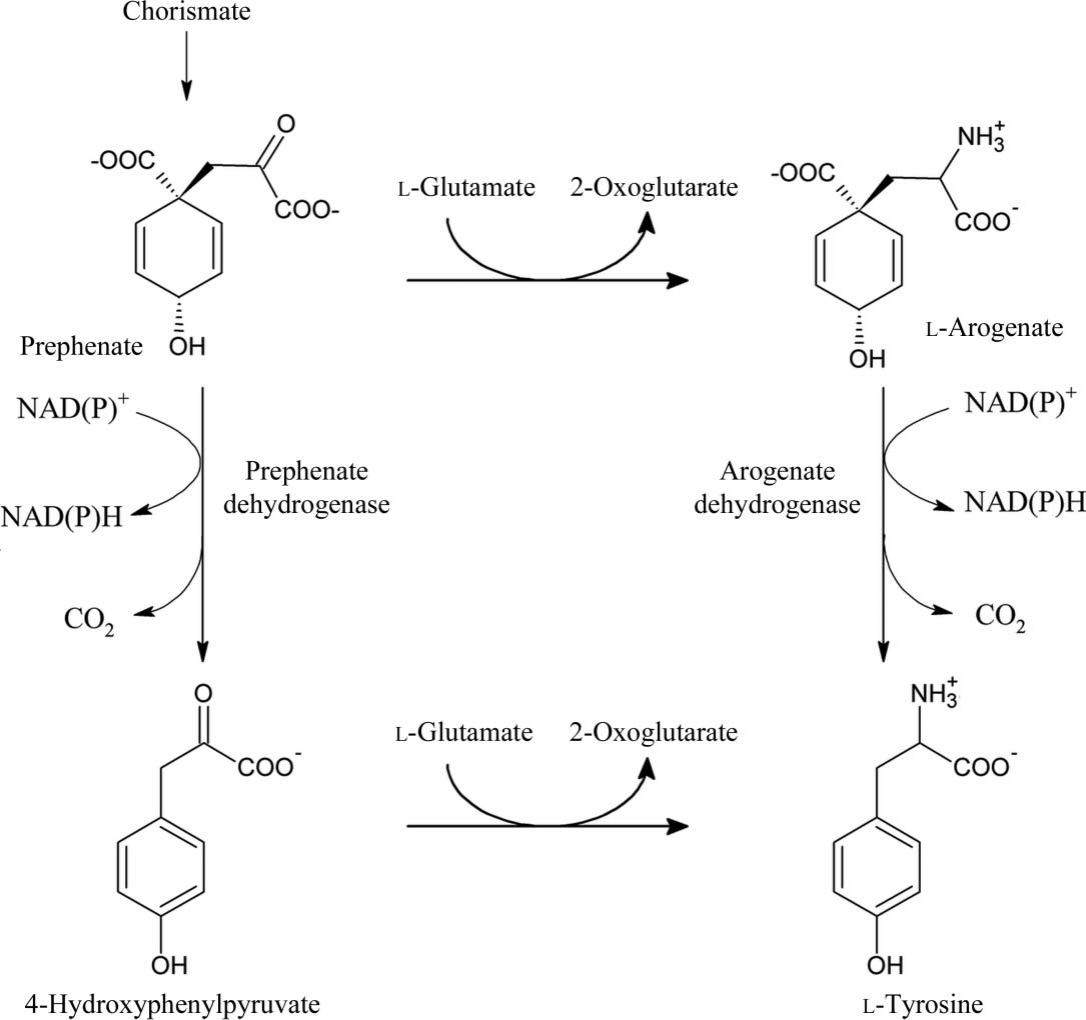
Tyrosine biosynthetic pathway. The figure is modified from The Enzyme Database (http://www.enzyme-database.org).

The TyrA protein family comprises dehydrogenases that are dedicated to l-tyrosine biosynthesis. These dehydrogenases can be classified into three groups according to their substrate specificity. Prephenate dehydrogenases (TyrAp or PDH) only use prephenate as a substrate, arogenate dehydrogenases (TyrAa of ADH) only accept arogenate and cyclohexadienyl dehydrogenases (TyrAc or CDH) use either prephenate or arogenate. The TyrA dehydrogenases convert prephenate to tyrosine through two different routes (Fig. 1 ▶). In the prephenate pathway, PDH enzymes catalyze the NAD(P)^+^-dependent oxidative decarboxylation of prephenate to 4-hydroxyphenyl­pyruvate (HPP), which is then converted to l-tyrosine by an aminotransferase. In the arogenate route, prephenate is first transaminated to l-arogenate by prephenate aminotransferase. l-Arogenate is then decarboxylated and converted to l-tyrosine by ADH. In addition to diverse substrate specificity, the TyrA family also exhibits diversity with respect to its cofactor specificity. TyrA proteins may be specific for NAD^+^ or NADP^+^ or may use both. TyrA proteins exist as either monofunctional or bifunctional proteins. The common fusion partners of TyrA proteins include chorismate mutase (*aro*Q; Calhoun *et al.*, 2001 ▶), 3-phosphoshikimate 1-carboxyvinyltransferase (*aro*F; Beller *et al.*, 2006 ▶) and an ACT (aspartate kinase–chorismate mutase–TyrA) regulatory domain (Chipman & Shaanan, 2001 ▶; Grant, 2006 ▶).

TyrAp is mostly present in low-GC Gram-positive organisms, such as *Bacillus subtilis*. TyrAa is abundant in higher plants and in at least three bacterial lineages, cyanobacteria, actinomycetes and *Nitrosomonas europaea*, whereas TyrAc is found in most bacteria. An analysis of the phylogenetic relationship of TyrA enzymes identified a distinct subgroup within the TyrAc group, denoted here as TyrAc_Δ (Song *et al.*, 2005 ▶). When the primary sequences of these TyrAc_Δ proteins are aligned with those of other TyrA groups, it is immediately apparent that the TyrAc_Δ proteins contain a number of deletions within the catalytic core region and possess a second functional domain, which classifies them as bifunctional enzymes. Biochemical studies have shown that this subgroup displays narrower substrate and cofactor specificity compared with the parent TyrAc enzymes. The TyrA enzymes from *Escherichia coli* and *Klebsiella pneumoniae* are the two best characterized TyrAc_Δ enzymes and both prefer prephenate over arogenate by more than one order of magnitude and only use NAD^+^ as cofactor (Ahmad & Jensen, 1987 ▶; Turnbull *et al.*, 1990 ▶). The studies further suggested that the TyrAc family, with its broad substrate specificity, represents the ancestral enzymes from which the TyrAc_Δ, TyrAa and TyrAp enzymes have evolved to exhibit a narrower range of substrate specificity (Song *et al.*, 2005 ▶).

The regulation of TyrA activity is important as prephenate is a common precursor in the biosynthesis of tyrosine and phenylalanine. TyrA enzymes are regulated by various mechanisms, including feedback inhibition and gene regulation by the Tyr operon (Cobbett & Delbridge, 1987 ▶). Kinetic studies of chorismate mutase/prephenate dehydrogenase (CM/PDH) from *E. coli* have led to the proposal of two different types of mechanism for tyrosine inhibition. Christopherson (1985 ▶) concluded that tyrosine acts as a competitive inhibitor in the de­hydrogenase reaction, whereas Turnbull, Morrison *et al.* (1991 ▶) suggested that tyrosine binds at a distinct allosteric site. *B. subtilis* PDH is inhibited competitively by tyrosine and non­competitively by tryptophan and HPP (Champney & Jensen, 1970 ▶). *B. subtilis* PDH has a C-terminal fusion of an ACT regulatory domain. The ACT domain was first identified in 1995 and is a small-molecule binding domain that is found in enzymes involved in amino-acid metabolism and transcription regulation. Small-molecule binding to the ACT domain is thought to control the enzyme activity through allosteric regulation. Thus, the noncompetitive inhibition by tryptophan and HPP in *B. subtilis* PDH might be a consequence of the presence of the ACT domain. Crystal structures of PDH enzymes from our study and from *Aquifex aeolicus* (Sun *et al.*, 2009 ▶) both revealed bound tyrosine, but only at the active site, which supports the role of tyrosine as a competitive inhibitor. The source of tyrosine in these two structures originated from protein expression and co­crystallization, respectively. Not all TyrA enzymes are inhibited by tyrosine; *Synechocystis* ADH, for example, is completely insensitive to competitive inhibition by tyrosine (Legrand *et al.*, 2006 ▶). Studies on *A. aeolicus* PDH (Sun *et al.*, 2009 ▶) showed that His217 is critical for the inhibitory effect of tyrosine where a His217Ala mutation completely abolished the inhibitory effect of tyrosine. Studies on *E. coli* TyrA reported similar results, in which a His257Ala mutation (His257 is equivalent to His217 in *A. aeolicus* PDH) abolished inhibition by tyrosine (Christendat *et al.*, 1998 ▶). Comparison of the crystal structure of *Synechocystis* ADH with that of *A. aeolicus* PDH revealed that Val182 is present in this location, which could account for the loss of tyrosine inhibition.

Studies of the enzymatic mechanisms of TyrA enzymes have revealed some variations in the reaction pathway. The kinetic data for *Synechocystis* ADH suggested a sequential substrate-binding event in which arogenate first binds to the protein, followed by the cofactor (Beller *et al.*, 2006 ▶), whereas kinetic studies on *E. coli* and *Arabidopsis thaliana* suggested a random addition of NAD^+^ and prephenate (Sampathkumar & Morrison, 1982 ▶; Rippert & Matringe, 2002 ▶). Kinetic studies using ^13^C-labeled substrates suggested a mechanism in which decarboxylation and proton transfer occur in a concerted manner (Hermes *et al.*, 1984 ▶). Kinetic and mutagenesis studies of several systems have identified key active-site residues (Christendat *et al.*, 1998 ▶; Christendat & Turnbull, 1999 ▶). Conserved histidine, arginine and serine residues are critical for enzyme activity. For instance, in *E. coli* TyrA His197 has been proposed to facilitate hydride transfer from prephenate to NAD^+^ by polarizing the 4-OH group of prephenate, whereas Arg294 is critical for substrate binding. A His197Ala mutation decreased the dehydrogenase activity significantly and an Arg294Gln mutation greatly increased the *K*_m_, but did not affect the *k*_cat_. In *A. aeolicus* PDH, Ser126 is hydrogen bonded to the 4′-OH of tyrosine and to NAD^+^ and could participate in both catalysis and ligand binding. The Ser126Ala mutation reduced *k*_cat_ 15-­fold and increased *K*_m_ tenfold.

The TyrA gene from *Haemophilus influenza* Rd KW20 encodes a bifunctional enzyme, chorismate mutase (EC 5.4.99.5)/prephenate dehydrogenase (EC 1.3.1.12) (CM/PDH), with a molecular weight of 43 kDa (residues 1–377) and a calculated isoelectric point of 5.56. The crystal structure of the prephenate dehydrogenase component (*Hinf*PDH; residues 81–377) of this TyrA enzyme was determined in complex with tyrosine and NAD^+^ at 2.0 Å resolution and represents the first PDH structure from a bifunctional TyrA enzyme. The structure was determined using the semi-automated high-throughput pipeline of the Joint Center for Structural Genomics (JCSG; Lesley *et al.*, 2002 ▶) as part of the National Institute of General Medical Sciences’ Protein Structure Initiative (PSI; http://www.nigms.nih.gov/Initiatives/PSI/).

## 2. Materials and methods

### 2.1. Protein production and crystallization

Clones were generated using the Polymerase Incomplete Primer Extension (PIPE) cloning method (Klock *et al.*, 2008 ▶). The gene encoding *Hinf*PDH (GenBank AAC22939, gi|1574749; Swiss-Prot TYRA) was amplified by polymerase chain reaction (PCR) from *H. influenzae* Rd KW20 genomic DNA using *PfuTurbo* DNA polymerase (Stratagene) and I-PIPE (Insert) primers (forward primer, 5′-­ctgtacttccagggcATGCGTGAATCCTATGCCA­ATGAAAACC-3′; reverse primer, 5′-aattaagtcgcgttaGCATAAAACGGCGTAGAA­CATCTTCAAT-3′; target sequence in upper case) that included sequences for the predicted 5′ and 3′ ends. The expression vector pSpeedET, which encodes an amino-terminal tobacco etch virus (TEV) protease-cleavable expression and purification tag (MG­SDKIHHHHHHENLYFQ/G), was PCR-amplified with V-PIPE (Vector) primers. The V-PIPE and I-PIPE PCR products were mixed to anneal the amplified DNA fragments together. *E. coli* GeneHogs (Invitrogen) competent cells were transformed with the V-PIPE/I-­PIPE mixture and dispensed onto selective LB–agar plates. The cloning junctions were confirmed by DNA sequencing. Using the PIPE method, the part of the gene encoding residues Met1–Phe80 was excluded from the final construct. Expression was performed in selenomethionine-containing medium with suppression of normal methionine synthesis (Van Duyne *et al.*, 1993 ▶). At the end of fermentation, lysozyme was added to the culture to a final concentration of 250 µg ml^−1^ and the cells were harvested and frozen. After one freeze–thaw cycle, the cells were sonicated in lysis buffer [50 m*M* HEPES pH 8.0, 50 m*M* NaCl, 10 m*M* imidazole, 1 m*M* tris(2-­carboxyethyl)phosphine–HCl (TCEP)] and the lysate was clarified by centrifugation at 32 500*g* for 30 min. The soluble fraction was passed over nickel-chelating resin (GE Healthcare) pre-equilibrated with lysis buffer, the resin was washed with wash buffer [50 m*M* HEPES pH 8.0, 300 m*M* NaCl, 40 m*M* imidazole, 10%(*v*/*v*) glycerol, 1 m*M* TCEP] and the protein was eluted with elution buffer [20 m*M* HEPES pH 8.0, 300 m*M* imidazole, 10%(*v*/*v*) glycerol, 1 m*M* TCEP]. The eluate was buffer-exchanged with TEV buffer (20 m*M* HEPES pH 8.0, 200 m*M* NaCl, 40 m*M* imidazole, 1 m*M* TCEP) using a PD-10 column (GE Healthcare) and incubated with 1 mg TEV protease per 15 mg of eluted protein. The protease-treated eluate was run over nickel-chelating resin (GE Healthcare) pre-equilibrated with HEPES crystallization buffer (20 m*M* HEPES pH 8.0, 200 m*M* NaCl, 40 m*M* imidazole, 1 m*M* TCEP) and the resin was washed with the same buffer. The flowthrough and wash fractions were combined and concentrated to 19.6 mg ml^−1^ by centrifugal ultrafiltration (Millipore) for crystallization trials. *Hinf*PDH was crystallized using the nanodroplet vapor-diffusion method (Santarsiero *et al.*, 2002 ▶) with standard JCSG crystallization protocols (Lesley *et al.*, 2002 ▶). Sitting drops composed of 200 nl protein mixed with 200 nl crystallization solution were equilibrated against a 50 µl reservoir at 293 K for 28 d prior to harvest. The crystallization reagent was composed of 0.04 *M* potassium dihydrogen phosphate, 20.0%(*v*/*v*) glycerol and 16.0%(*w*/*v*) PEG 8000. A rod-shaped crystal of approximate dimensions 0.1 × 0.05 × 0.05 mm was harvested for data collection. No additional cryoprotectant was added to the crystal. Initial diffraction screening was carried out using the Stanford Automated Mounting system (SAM; Cohen *et al.*, 2002 ▶) at the Stanford Synchrotron Radiation Lightsource (SSRL, Menlo Park, California, USA). The crystal was indexed in the tetragonal space group *P*4_1_2_1_2. The oligomeric state of *Hinf*PDH was determined to be a dimer by gel filtration using a 0.8 × 30 cm Shodex Protein KW-803 column (Thomson Instruments) equilibrated in 20 m*M* Tris, 200 m*M* NaCl, 0.5 m*M* TCEP pH 7.5 and pre-calibrated with gel-filtration standards (Bio-Rad). Protein con­centrations were determined using the Coomassie Plus assay (Pierce).

### 2.2. Data collection, structure solution and refinement

Multiple-wavelength anomalous diffraction (MAD) data were collected on beamline 11-1 at the SSRL using a 0.1 × 0.1 mm X-ray beam at wavelengths corresponding to the inflection (λ_1_) and remote (λ_2_) wavelengths of a selenium MAD experiment. The data sets were collected at 100 K using a Rayonix MAR Mosaic MX-325 CCD detector. The MAD data were integrated and reduced using *XDS* and scaled with the program *XSCALE* (Kabsch, 1993 ▶, 2010*a* ▶,*b* ▶). An initial substructure solution was obtained with *SHELXD* (Sheldrick, 2008 ▶) and the phases were refined using *autoSHARP* (Vonrhein *et al.*, 2007 ▶), which gave a mean figure of merit of 0.48 with 14 selenium sites. Automated model building was performed with *ARP*/*wARP* (Cohen *et al.*, 2004 ▶). Model completion and refinement were per­formed with *Coot* (Emsley & Cowtan, 2004 ▶) and *REFMAC* 5.2 (Winn *et al.*, 2003 ▶) using the high-energy remote (λ_2_) data set. The refinement included phase restraints from *SHARP* and TLS refinement with three TLS groups per chain. *CCP*4 programs were used for data conversion and other calculations (Collaborative Computational Project, Number 4, 1994 ▶).

During structure refinement, additional electron density was found at the active site. The density was well defined and could be unambiguously assigned to a tyrosine molecule and an NAD^+^ cofactor. As these molecules were not added during the crystallization experiment, they must have been acquired during protein expression and have remained bound during purification and crystallization. Data-collection and refinement statistics are summarized in Table 1 ▶.

### 2.3. Validation and deposition

**Figure 2:**
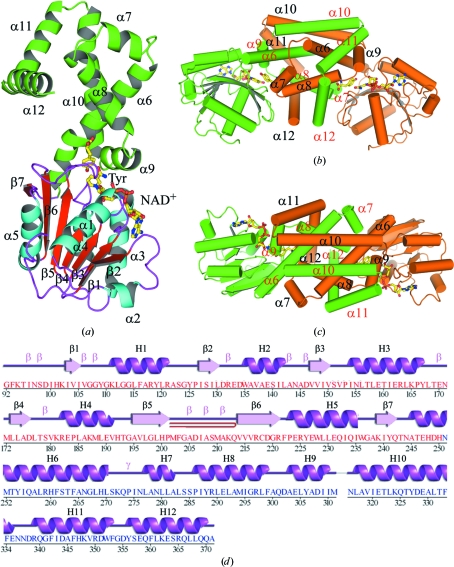
Structure of *Hinf*PDH. (*a*) Ribbon diagram of a *Hinf*PDH monomer complexed with NAD^+^ and a tyrosine molecule. Helices α1–α12 and strands β1–β7 are indicated. The C-terminal domain is colored green and the helices and β-strands of the N-terminal domain are colored cyan and red, respectively. Bound tyrosine and NAD^+^ molecules are shown in ball-and-stick representation; C, O, N and phosphate atoms are colored yellow, red, blue and orange, respectively. (*b*) Ribbon diagram of the *Hinf*PDH dimer; monomer *A* is colored green and monomer *B* is colored orange. Helices are shown as cylinders. (*c*) Top view compared with (*b*) of the *Hinf*PDH dimer. (*d*) Diagram showing the secondary-structure elements of *Hinf*PDH superimposed on its primary sequence. The labeling of secondary-structure elements is in accord with *PDBsum* (http://www.ebi.ac.uk/pdbsum), where α-helices (H1–H12) and β-strands (β1–β7) are sequentially labeled, β-turns and γ-turns are designated by Greek letters (β, γ) and β-hairpins are indicated by red loops.

The quality of the crystal structure was analyzed using the *JCSG Quality Control* server (http://smb.slac.stanford.edu/jcsg/QC). This server verifies the stereochemical quality of the model using *AutoDepInputTool* (Yang *et al.*, 2004 ▶), *MolProbity* (Chen *et al.*, 2010 ▶) and *WHATIF* v.5.0 (Vriend, 1990 ▶), the agreement between the atomic model and the data using *SFCHECK* v.4.0 (Vaguine *et al.*, 1999 ▶) and *RESOLVE* (Terwilliger, 2004 ▶), the protein sequence using *ClustalW* (Thompson *et al.*, 1994 ▶), the atom occupancies using *MOLEMAN*2 (Kleywegt, 2000 ▶) and the consistency of NCS pairs. Protein quaternary-structure analysis was conducted using the *PISA* server (http://www.ebi.ac.uk/msd-srv/prot_int/pistart.html; Krissinel & Henrick, 2005 ▶). Fig. 2 ▶(*d*) was adapted from *PDBsum* (Laskowski, 2009 ▶); all other figures were prepared with *PyMOL* (DeLano, 2002 ▶). Atomic coordinates and experimental structure factors have been deposited in the PDB under accession code 2pv7.

## 3. Results and discussion

### 3.1. Overall structure

The final model consists of a dimer of the PDH domain of TyrA (residues 92–371 for chains *A* and *B*), two nicotinamide adenine dinucleotides (NAD^+^), two tyrosines and 393 water molecules in the asymmetric unit (Figs. 2 ▶ *a*, 2 ▶ *b* and 2 ▶ *c*). No electron density was observed for Gly80–Phe91, Asp312–Glu315 and Asn372–Gly377 in chain *A* and Gly80–Val91 and Asn372–Gly377 in chain *B*. The side chains of Lys212 and Lys348 from chain *A* and of Arg132, Lys212, Lys239, Gln325 and Ala371 from chain *B* were omitted owing to poor electron density. The Matthews coefficient (*V*_M_; Matthews, 1968 ▶) was 2.98 Å^3^ Da^−1^ and the estimated solvent content was 58.7%. The Ramachandran plot produced by *MolProbity* showed that 98% of the residues are in favored regions, with no outliers.

Each monomer consists of an N-terminal α/β dinucleotide-binding domain (residues 92–243) and a C-terminal α-helical dimerization domain (residues 244–371) (Fig. 2 ▶ *a*). The active site is located at the domain interface. The N-terminal domain adopts a modified Rossmann fold, which consists of a parallel seven-stranded β-sheet (strand order β2-β1-β3-β4-β5-β6-β7) with the α1 helix on one face of the β-­sheet and the α2, α3, α4 and α5 helices on the other. Structural comparisons of *Hinf*PDH and other nucleotide-binding proteins, including other TyrA enzymes, show that *Hinf*PDH lacks the α–β structural motif between α2 and β2 that is present and is part of the β-­sheet in other nucleotide-binding proteins. The C-terminal domain consists of seven helices (α6–α12) that form the dimer interface; the helices from each monomer are intertwined into a tightly packed helical bundle with a buried surface of 11 000 Å^2^ (Figs. 2 ▶ *b* and 2 ▶ *c*). The two monomers are structurally similar to each other, with an r.m.s.d. of 0.2 Å for 268 equivalent C^α^ atoms.

### 3.2. The active site

**Figure 3:**
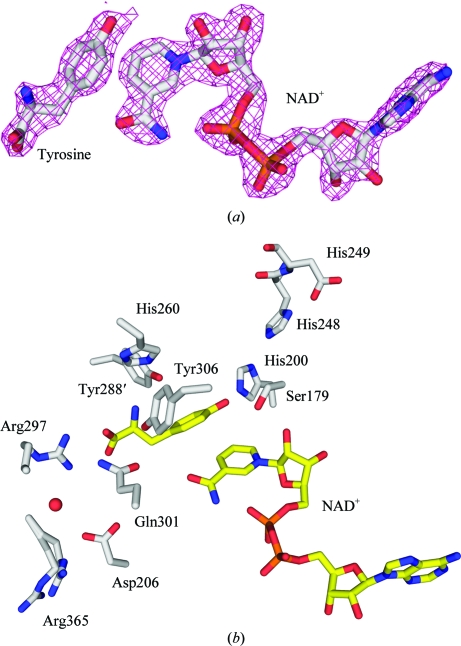
Active site of *Hinf*PDDH. (*a*) Final model of tyrosine and NAD^+^ molecules fitted in 2*F*_o_ − *F*_c_ electron-density maps prior to model building as output from the last step of *XSOLVE* (JCSG, unpublished work). The map is contoured at 1σ. (*b*) Active site of *Hinf*PDDH showing key active-site residues and the bound tyrosine and NAD^+^ molecules in ball-and-stick representation. C atoms are colored gray for the protein and yellow for the ligands; O, N and phosphate atoms are colored red, blue and orange, respectively.

The active site is located in the cleft between the N- and C-terminal domains (Figs. 2 ▶ *a*, 2 ▶ *b* and 2 ▶ *c*). One tyrosine and one NAD^+^ are bound in each monomer (Fig. 3 ▶ *a*). His200, Ser179 and Arg297 (Fig. 3 ▶ *b*) are among the important residues for enzyme catalysis and/or ligand binding and are conserved in TyrA enzymes across all kingdoms of life. The His200 imidazole is hydrogen bonded to the 4′-OH of the bound tyrosine. Ser179 hydrogen bonds to both the ribose O atom of nicotinamide nucleoside and the 4′-OH of the bound tyrosine and is important for orientating prephenate and NAD^+^ for catalysis. The Arg297 guanadinium forms a pair of electrostatic interactions with the tyrosine carboxyl, which also interacts with Gln301 from the α8 helix. The tyrosine amino group hydrogen bonds to Tyr306 from α9 and Tyr288′ from α8 of the adjacent monomer. His260 is located close to the tyrosine amino group and could be involved in regulation of tyrosine inhibition in a similar way to His217 in *A. aeolicus.*

The aromatic ring of the bound tyrosine packs against the nico­tinamide ring of NAD^+^ such that the 4′-OH of tyrosine is approximately 4 Å away from C4 of the nicotinamide ring. Assuming that prephenate adopts the same binding mode, the structure suggests that the hydride is transferred from prephenate to the *si* face of NAD^+^, which is consistent with a previous proton NMR study on the *E. coli* TyrA enzyme using isotope-labeled NAD-4-d (Hermes *et al.*, 1984 ▶).

The binding mode of NAD^+^ is similar to those of arogenate de­hydrogenase from *Synechocystis* sp. and prephenate dehydrogenase from *A. aeolicus* (Legrand *et al.*, 2006 ▶; Sun *et al.*, 2006 ▶). The pyrophosphate of NAD^+^ interacts with the P-loop (Gly108–Gly113) between α1 and β1, forming hydrogen bonds to Lys111 and the main-chain amides of Lys111 and Leu112. The diol of the adenylyl ribose hydrogen bonds to Asp131 and Lys132 from the loop located between α2 and β2. The adenine ring is sandwiched between Val152 and Arg132 from helices α2 and α3, with its N1 hydrogen bonded to Trp135 from α2. The diol of the nicotinamide ribose is hydrogen bonded to Ser179 and Val152. The nicotinamide ring interacts with the protein mainly through hydrophobic interactions.

### 3.3. Structural comparison with other TyrA enzymes

A search with *FATCAT* (Ye & Godzik, 2004 ▶) using the *Hinf*PDH coordinates identified the closest structural homologues of *Hinf*PDH in the PDB as the prephenate dehydrogenases from *A. aeolicus* (*Aaeo*PDH; PDB code 2g5c; Sun *et al.*, 2006 ▶) and *Streptococcus thermophilus* (*Sthe*PDH; PDB code 3dzb; Z. Zhang, S. Eswara­moorthy, S. K. Burley & S. Swaminathan, unpublished work) and the arogenate dehydrogenase from *Synechocystis* sp. (*Syne*ADH; PDB code 2f1k; Legrand *et al.*, 2006 ▶). *Hinf*PDH is bifunctional, whereas the other three enzymes are monofunctional. The pairwise sequence identities between *Hinf*PDH and *Aaeo*PDH, *Sthe*PDH and *Syne*ADH are 20, 27 and 25%, respectively. Despite the low sequence identity, the overall structures of these enzymes are very similar. The structures of *Aaeo*PDH complexed with the ligands NAD^+^ (PDB code 2g5c), NAD^+^ and l-tyrosine (PDB code 3ggg), NADH and 4-hydroxylphenylpyruvate (HPP; PDB code 3ggo) and NAD^+^ and 4-hydroxyphenylpropionate (PDB code 3ggp) are available (Sun *et al.*, 2009 ▶), but only the first two structures were used for comparison because they are sufficient to represent the two unique enzyme states; they are denoted *Aaeo*PDH and *Aaeo*PDH–Tyr–NAD^+^, respectively. *Syne*ADH has an NADP^+^ bound at the active site and *Sthe*PDH has no ligand bound. The dehydrogenase activity of *Syne*ADH is strictly dependent on arogenate and NADP^+^ (Legrand *et al.*, 2006 ▶). Conversely, NAD^+^ and prephenate are the preferred cofactor and substrate for *Aaeo*PDH, although a very low level of dehydrogenase activity is detected when NADP^+^ with pre­phenate or NAD^+^ with arogenate are used (Bonvin *et al.*, 2006 ▶).

**Figure 4:**
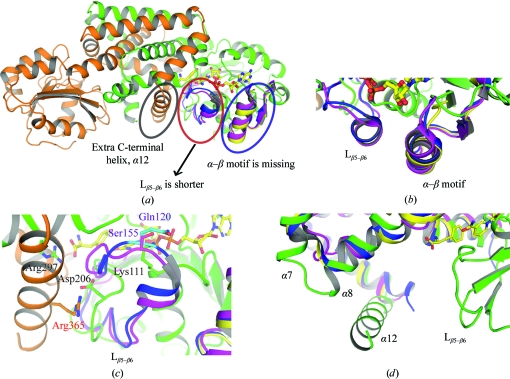
Structural differences between *Hinf*PDH and its structural homologues. (*a*) Superimposition of *Hinf*PDH, *Aaeo*PDH, *Sthe*PDH and *Syne*ADH reveals unique structural differences in* Hinf*PDH including a missing α–β motif, a shorter L_β5–β6_ loop, and an extra C-terminal helix. *Hinf*PDH is colored green or orange for each monomer. The *Aaeo*PDH, *Sthe*PDH and *Syne*ADH structures are colored magenta, yellow and blue, respectively. Bound tyrosine and NAD^+^ molecules are shown in ball-and-stick representation. (*b*) Enlarged view of the area around the α–β motif showing that *Hinf*PDH is missing the α–β motif compared with *Aaeo*PDH, *Sthe*PDH and *Syne*ADH. (*c*) Enlarged view of the area around L_β5–β6._ Lys111 of *Hinf*PDH is shown with a green backbone, Ser155 of *Aaeo*PDH is in magenta and Gln120 of *Syne*ADH is in blue. (*d*) Enlarged view of the C-terminal area showing that an extra C-terminal helix is present in *Hinf*PDH. The α7 and α8 helices of *Hinf*PDH superimposed onto structurally equivalent helices in *Aaeo*PDH, *Sthe*PDH and *Syne*ADH are also indicated.

**Figure 5:**
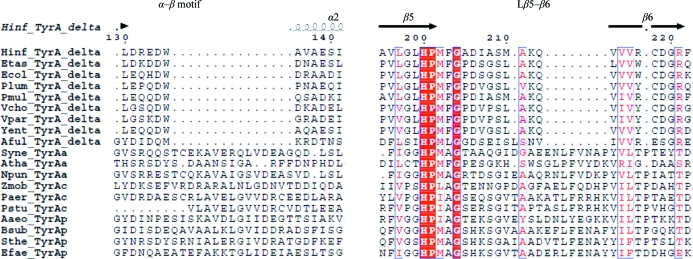
Multiple sequence alignment of representative TyrA enzymes. Regions around the α–β motif and L_β5–β6_ are shown. Abbreviations: Hinf, *Haemophilus influenzae* Rd KW20; Etas, *Erwinia tasmaniensis* Et1/99; Ecol, *Escherichia coli* str. K-12 substr. MG1655; Plum, *Photorhabdus luminescens* subsp. *laumondii* TTO1; Pmul, *Pasteurella multocida* subsp. *multocida* str. Pm70; Vcho, *Vibrio cholerae* O1 biovar eltor str. N16961; Vpar, *Vibrio parahaemolyticus* RIMD 2210633; Yent, *Yersinia enterocolitica* subsp. *enterocolitica* 8081; Aful, *Archaeoglobus fulgidus*; Syne, *Synechocystis* sp. (Legrand *et al.*, 2006 ▶); Atha, *Arabidopsis thaliana* (Rippert & Matringe, 2002 ▶); Npun, *Nostoc punctiforme* PCC 73102 (Song *et al.*, 2005 ▶); Zmob, *Zymomonas mobilis* (Zhao *et al.*, 1993 ▶); Paer, *Pseudomonas aeruginosa* PA7 (Xia & Jensen, 1990 ▶); Pstu, *Pseudomonas stutzeri* (Xie *et al.*, 2000 ▶); Aaeo, *Aquifex aeolicus* VF5 (Bonvin *et al.*, 2006 ▶); Bsub, *Bacillus subtilis* (Champney & Jensen, 1970 ▶); Sthe: *Streptococcus thermophilus* LMG 18311 (Song *et al.*, 2005 ▶); Efae, *Enterococcus faecalis* V583 (Song *et al.*, 2005 ▶).

Although the overall fold is similar, structural comparisons revealed important differences around the active site. Compared with other TyrA enzymes, *Hinf*PDH lacks an α–β structural motif between α2 and β2, the loop between β5 and β6 (L_β5–β6_) is much shorter, and an extra helix α12 is found at the C-terminus (Fig. 4 ▶). Multiple sequence alignment of many representative TyrA proteins clearly shows that the α–β motif and L_β5–β6_ represent unique structural differences between bifunctional and monofunctional TyrA enzymes (Fig. 5 ▶), but the extra C-terminal helix is less obviously discernable from the sequence comparisons, presumably because the exact end point of the prephenate dehydrogenase domain is difficult to determine for cases such as TyrA-*aro*F or TyrA-ACT fusions in which *aro*F and ACT are fused at the C-terminal end.

In *Hinf*PDH, L_β5–β6_ is eight residues shorter than in *Aaeo*PDH, *Sthe*PDH and *Syne*ADH. This loop is well ordered in the *Hinf*PDH, *Aaeo*PDH and *Syne*ADH structures (Fig. 4 ▶ *c*) but is disordered in the *Sthe*PDH structure owing to the absence of bound cofactor. In *Aaeo*PDH and *Syne*ADH, this loop extends along the cofactor-binding site. Ser155 in *Aaeo*PDH and Gln120 in *Syne*ADH are in structurally equivalent positions (Fig. 4 ▶ *c*) and both form hydrogen bonds to the pyrophosphate O atom of the bound cofactor. However, in *Hinf*PDH no residue is structurally equivalent because of the shorter loop and Lys111 from α1 instead provides the equivalent interaction with the pyrophosphate O atom (Fig. 4 ▶ *c*). The equivalent residues to Lys111 in *Aaeo*PDH and *Syne*ADH are Phe40 and Leu10, respectively, and neither side chain can form hydrogen bonds to the pyrophosphate. This suggests that although a shorter L_β5–β6_ loop has evolved in *Hinf*PDH, the ability to bind cofactor is not affected.

In *Syne*ADH, the phosphate group of the adenosine ribose of NADP^+^ is recognized by helical residues in the α–β motif. The phosphate group is stabilized by electrostatic interaction with Arg31 and hydrogen-bonding interactions with Gln32 and Thr35, as well as with the main chain of Arg31 and Gln32. The equivalent residues in *Aaeo*PDH are Ile63, Asn64 and Ser67. A loss of electrostatic interaction caused by the substitution of Arg by Ile may explain why *Aaeo*PDH prefers NAD^+^ over NADP^+^ as cofactor. In *Sthe*PDH, the equivalent residues are Arg36, Ser37 and Ser40. Thus, the ability to form electrostatic and hydrogen-bonding interactions is similar to that of *Syne*ADH, suggesting that *Sthe*PDH is capable of binding NADP^+^. In *Hinf*PDH, Arg132 is structurally equivalent to *Syne*ADH Arg31, but residues equivalent to Gln32 and Thr35 are absent owing to the lack of the α–β structural motif. It is possible that *Hinf*PDH also prefers NAD^+^ over NADP^+^ in a manner similar to bifunctional TyrA enzymes from *E. coli* and* K. pneumonia*.

A smaller local difference among these TyrA structures is the loop joining α7 and α8 and the adjacent residues (Fig. 4 ▶ *d*). This loop takes a wider turn in *Hinf*PDH compared with that in *Aaeo*PDH, *Sthe*PDH and *Syne*ADH and is positioned next to α12. Ser284–Leu290 in this region are highly conserved in TyrAc_Δ sequences, suggesting that they play important roles.

### 3.4. Global conformational change

**Figure 6:**
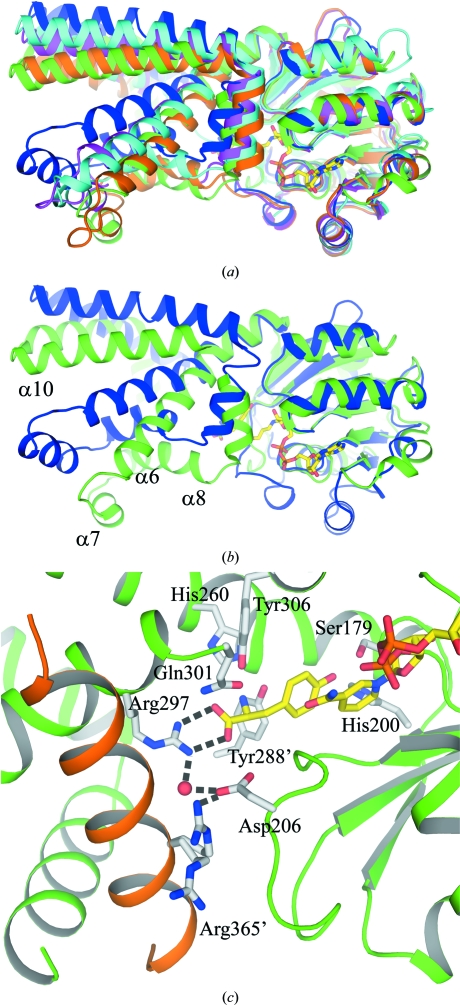
Superimposition of TyrA structures showing differences in the relative orientation of the N- and C-terminal domains and a close-up view of the active site of *Hinf*PDH. (*a*) Superimposition of *Hinf*PDH, *Aaeo*PDH, *Aaeo*PDH–Tyr–NAD^+^, *Sthe*PDH and *Syne*ADH reveals differences in the relative orientation of the N- and C-terminal domains in these TyrA proteins. *Hinf*PDH, *Aaeo*PDH, *Aaeo*PDH (monomer *B*)–Tyr–NAD^+^, *Sthe*PDH and *Syne*ADH are colored green, magenta, orange, cyan and blue, respectively. (*b*) Same orientation as (*a*); for clarity, only *Hinf*PDH and *Syne*ADH are shown. (*c*) An ionic network in the active site of *Hinf*PDH consists of Arg297, a bridging water molecule, Asp206 and Arg365′ from adjacent molecule in a dual conformation. His260, Tyr288, Gln301 and Tyr306 that could be involved in substrate selectivity are also shown. Hydrogen bonds are indicated as dashed lines.

Another important difference between *Hinf*PDH and other TyrA structures is the relative orientation of the respective N- and C-­terminal domains. Pairwise structural alignment of *Hinf*PDH with *Aaeo*PDH, *Aaeo*PDH–Tyr–NAD^+^, *Sthe*PDH and *Syne*ADH using only the N-terminal domain gives r.m.s.d.s of 1.8, 1.7, 1.9 and 1.5 Å, respectively, for 128–132 superimposed C^α^ atoms (0.8–0.9 Å for core β-sheet residues). Upon structural superimposition, it is immediately noticeable that differences in the relative orientation of the N- and C-­terminal domains are present in these TyrA structures (Figs. 6 ▶ *a* and 6 ▶ *b*). A hinge region around Glu242–Asn244 connects the N- and C-­terminal domains at the domain interface opposite to the substrate-binding site. Superimposition using the C-terminal domain gives similar results, although the results are less obvious owing to internal structural differences within the C-terminal domains of these TyrA structures. Therefore, the discussion below is based on superimpositions using the N-terminal domain.

Using the C-terminal domain of *Hinf*PDH as a reference, the C-­terminal domains of *Aaeo*PDH, *Sthe*PDH and *Syne*ADH are farther away from the substrate-binding site; therefore, *Hinf*PDH represents the most closed form, *Syne*ADH is the most open form and *Aaeo*PDH and *Sthe*PDH are in intermediate states (Figs. 6 ▶ *a* and 6 ▶ *b*). Monomer *B* of *Aaeo*PDH–Tyr–NAD^+^ has both NAD^+^ and tyrosine bound and the conformation is closed, similar to *Hinf*PDH. Monomer *A* of *Aaeo*PDH–Tyr–NAD^+^ only has NAD^+^ bound and the conformation is similar to *Aaeo*PDH. Comparison of the *Aaeo*PDH and *Aaeo*PDH–Tyr–NAD^+^ structures suggest that tyrosine induces a conformational change upon binding. Hence, it is possible that the binding of tyrosine to *Hinf*PDH also induces a conformational change from an open to a closed form and the closed form is captured in the current *Hinf*PDH structure. A crystal structure of apo *Hinf*PDH could provide direct evidence for this proposal. In the closed conformation, α6, α8, α9 and α8′ (α8 from monomer *B*) are close to the bound tyrosine and α8 and α12′ (α12 from monomer *B*) are near L_β5–β6_, facilitating closure of the active site and the proper alignment of active-site residues for catalysis. Key active-site residues in this region include His260 (α6), Arg297, Gln301 (α8), Tyr288′ (α8′), Tyr306 (α9), Arg365′ (α12′) and Asp206 (L_β5–β6_). If a tyrosine molecule is modeled adjacent to the cofactor in *Aaeo*PDH and *Sthe*PDH, the residue equivalent to Arg297 of *Hinf*PHDH is too distant to interact with the tyrosine. The conformational change is independent of cofactor binding as the structures of *Syne*ADH with NADP^+^ bound, *Aaeo*PDH with NAD^+^ bound and *Sthe*PDH without any cofactor are all in similar open conformations.

In the closed conformation of *Hinf*PDH an ionic network con­sisting of Arg297, a bridging water molecule, Asp206 from L_β5–β6_ and Arg365′ from α12′ is observed that may be involved in gating the active site (Fig. 6 ▶ *c*). The bridging water is present in both monomers. The Arg365′ side chain adopts dual conformations, with one con­formation participating in the ionic network and the other pointing away from the active site. This dual conformation may be part of the gating mechanism, in which an ionic network forms on closure of the active site after substrate is bound and is broken when the product is released. It is worth noting that Asp206 is absolutely conserved and Arg365 is highly conserved in chorismate mutase/prephenate dehydrogenase sequences but not in monofunctional TyrA enzymes. Thus, the active-site gating mechanism might be different in bifunctional and monofunctional TyrA enzymes.

### 3.5. Insights into the catalytic mechanism

Previous studies on the pH-dependence of the *E. coli* TyrA enzyme showed that a catalytic group with a p*K*_a_ value of about 6.5 is de­protonated for dehydrogenase activity (Turnbull, Cleland *et al.*, 1991 ▶). Subsequent site-directed mutagenesis experiments revealed that this critical catalytic residue is His197 (Christendat *et al.*, 1998 ▶). The catalytic mechanism of the oxidative decarboxylation of *E. coli* TyrA was investigated and suggested a concerted mechanism in which hydride transfer and decarboxylation occur in a concerted manner. It was proposed that His197 provides the driving force for the dehydrogenase reaction by polarizing the 4′-hydroxyl group of prephenate (Christendat *et al.*, 1998 ▶). It was also postulated that since the end-product of the reaction is aromatic, polarization of the 4′-OH group is sufficient to lower the energy barrier for the reaction, rather than deprotonation of the 4′-OH group to form a β-keto acid intermediate in a stepwise mechanism. The crystal structure of *Hinf*PDH shows that the N^∊2^ atom of His200 (equivalent to His197 in *E. coli* TyrA) is hydrogen bonded to the 4′-hydroxyl group of tyrosine at a distance of ∼2.6 Å. A hydrogen-bonding network between His200, His248 and Asp249 is observed in which His200 N^δ1^ hydrogen bonds to His248 N(H)^∊2^ and His248 N(H)^δ1^ hydrogen bonds to Asp249 O^δ1^. Asp249 is located near the protein surface. As for His200, His248 and Asp249 are highly conserved in TyrA sequences. This hydrogen-bonding network can help to maintain His200 N^∊2^ in a deprotonated state. The Ser179 hydrogen bond to the 4′-OH of the bound tyrosine can provide an additional driving force for the reaction by polarizing the 4′-OH group since the equivalent Ser126 in *Aaeo*PDH is critical for catalysis. In addition, *Hinf*PDH and *E. coli* TyrA share 57% sequence identity in their prephenate dehydro­genase domains and all key active-site residues are conserved. Hence, *Hinf*PDH is likely to adopt a concerted mechanism for dehydro­genase reaction as found for *E. coli* TyrA. In *Hinf*PDH, tyrosine is bound directly at the catalytic site, suggesting that it acts as a competitive inhibitor.

How TyrA enzymes evolved to be specific for prephenate or arogenate is intriguing because prephenate and arogenate have very similar structures. In *Hinf*PDH, Gln301, Tyr306, Tyr288 and His260 are positioned close to the amino and carboxyl groups of the bound tyrosine and could be involved in substrate specificity (Fig. 6 ▶ *c*). Gln301, Tyr306 and Tyr288 are highly conserved in bifunctional TyrA sequences. In other TyrA sequences, Gln301 is replaced by Gly, Ser or Thr. Tyr306 is relatively conserved as Trp in TyrAp and TyrAc, but is Trp, Gly or Val in TyrAa. Tyr288 is located at the N-terminus of α8, where some local structural differences are found between *Hinf*PDH and other TyrA structures. Alignment of TyrA sequences shows a gap of approximately four residues around Tyr288 in the TyrAp, TyrAa and TyrAc sequences. His260 is conserved in TyrA, except for some TyrAas, where it is Val or Gln. Given the structural resemblance between prephenate and arogenate, further experiments to elucidate the exact prephenate-binding mode will advance our understanding of substrate specificity in TyrA enzymes.

## 4. Conclusions

*Hinf*PDH is the first prephenate dehydrogenase structure to be determined from a bifunctional TyrA enzyme. This structure reveals active-site residues that are important for catalysis and/or ligand binding and are consistent with previously determined structures of other TyrA enzymes. The comparison of *Hinf*PDH with other known TyrA structures indicates important differences that appear to be characteristic features that differentiate the bifunctional and monofunctional TyrA enzymes and suggest that the regulation of enzyme activity is likely to differ between bifunctional and monofunctional TyrA enzymes. These structural differences may be related to the presence of a chorismate mutase domain in the bifunctional TyrA enzymes. A crystal structure of full-length *H. influenzae* TyrA should provide insight into this question. Additional information about the proteins described in this study is available from TOPSAN (Krishna *et al.*, 2010 ▶) at http://www.topsan.org/explore?PDBid=2pv7.

## Supplementary Material

PDB reference: chorismate mutase/prephenate dehydrogenase, 2pv7

## Figures and Tables

**Table 1:** Summary of crystal parameters, data-collection and refinement statistics for *Hinf*PDH (PDB code 2pv7) Values in parentheses are for the highest resolution shell.

	λ_1_ MADSe	λ_2_ MADSe
Space group	*P*4_1_2_1_2	
Unit-cell parameters (Å)	*a* = *b* = 127.79, *c* = 100.62	
Data collection		
Wavelength (Å)	0.9792	0.9184
Resolution range (Å)	29.7–2.00 (2.07–2.00)	29.7–2.00 (2.07–2.00)
No. of observations	411204	412324
No. of unique reflections	56589	56593
Completeness (%)	99.6 (98.8)	99.7 (99.3)
Mean *I*/σ(*I*)	12.4 (2.5)	12.9 (2.6)
*R*_merge_ on *I*† (%)	6.3 (51.5)	6.2 (50.7)
*R*_meas_ on *I*‡ (%)	7.3 (60.5)	7.2 (59.5)
Model and refinement statistics		
Data set used in refinement	λ_2_ MADSe	
Resolution range (Å)	29.7–2.00	
Cutoff criterion	|*F*| > 0	
No. of reflections (total)	56541	
No. of reflections (test)	2870	
Completeness	99.8	
*R*_cryst_§	0.161	
*R*_free_¶	0.194	
Stereochemical parameters		
Restraints (r.m.s.d. observed)		
Bond angles (°)	1.61	
Bond lengths (Å)	0.017	
Average isotropic *B* value (Å^2^)	35.4††	
ESU‡‡ based on *R*_free_ (Å)	0.12	
Protein residues/atoms	556/4427	
Water molecules/ligands	393/4	

† *R*_merge_ = 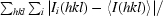 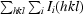.

‡ *R*_meas_ = 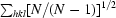 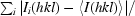 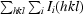 (Diederichs & Karplus, 1997 ▶).

§ *R*_cryst_ = 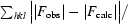 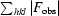, where *F*_calc_ and *F*_obs_ are the calculated and observed structure-factor amplitudes, respectively.

¶ *R*_free_ is the same as *R*_cryst_ but for 5.1% of the total reflections that were chosen at random and omitted from refinement.

†† This value represents the total *B* that includes TLS and residual *B* components.

‡‡ The estimated overall coordinate error (Collaborative Computational Project, Number 4, 1994 ▶; Cruickshank, 1999 ▶).
